# Preliminary Study on the Effect and Molecular Mechanism of Tetrandrine in Alleviating Pulmonary Inflammation and Fibrosis Induced by Silicon Dioxide

**DOI:** 10.3390/toxics11090765

**Published:** 2023-09-09

**Authors:** Yi Wang, Bin Cheng, Yu-Jia Lin, Rui Wang, Jie Xuan, Hai-Ming Xu

**Affiliations:** 1 School of Public Health, Ningxia Medical University, Yinchuan 750004, China; 2 The Key Laboratory of Environmental Factors and Chronic Disease Control of Ningxia, Yinchuan 750004, China; 3 The Fifth People’s Hospital of the Ningxia Hui Autonomous Region, Shizuishan 753000, China

**Keywords:** silicosis, tetrandrine, SiO_2_, lung inflammation, pulmonary fibrosis, autophagy

## Abstract

This study aims to explore the molecular mechanism of tetrandrine (Tet) in alleviating pulmonary inflammation and fibrosis induced by silica (SiO_2_) from the perspective of autophagy. C57BL/6J mice were selected as experimental animals, and SiO_2_ was exposed by intranasal instillation. Tet was intervened by oral gavage. The mice were euthanized on the 7th and 42nd day of SiO_2_ exposure, and lung tissues were collected for histopathological, molecular biological, immunological, and transmission electron microscopy analysis. The results showed that SiO_2_ exposure could lead to significant lung inflammation and fibrosis, while Tet could significantly reduce SiO_2_ exposure-induced lung inflammation and fibrosis. Molecular mechanism research indicated that, compared with SiO_2_ expose group, Tet intervention could significantly reduce the expression levels of inflammatory cytokines and fibrosis markers (TNF-α, IL-1β, MCP-1, TGF-β1, HYP, Col-I, and Fn), and regulate the expression of key molecules ATG7, microtubule-associated protein 1 light chain 3B (LC3B), and P62 in the autophagy pathway to improve the blocking of autophagic flux, promote the recovery of autophagic lysosomal system function, and inhibit apoptosis. In summary, Tet can alleviate silica-induced lung inflammation and fibrosis, which may be achieved by regulating the expression of key molecules in the autophagy process and associated apoptotic pathway.

## 1. Introduction

Silicosis is a serious occupational lung disease caused by long-term inhalation of dust containing free SiO_2_, which is mainly characterized by sustained inflammation and irreversible fibrosis [1]. It remains one of the most dangerous occupational diseases in the world, with more than 10,000 people dying of silicosis every year [2]. Nevertheless, there is no unique medicine that may cure silicosis overall because of its complex pathophysiology, difficulty in early diagnosis, and poor prognosis. Consequently, the prevention and control of occupational diseases in China and around the world continue to be focused on the prevention and control of silicosis. Wet work and wearing dust protection are two protective measures that have been implemented to reduce the range of diseases linked to SiO_2_ exposure, but new cases are still constantly emerging in industries like brick manufacturing, mining, and construction where silica materials are frequently used. However, there has been little progress in the development of alternative therapeutic modalities to pulmonary transplantation so far [3].

Macrophages, as functional cells of the innate immune system, are able to phagocytose foreign bodies, dead cells, and debris, and release a variety of inflammatory factors (TNF-α, IL-1β, IL-6, etc.) and chemokines (MCP-1, MIP-1α, MIP-1β, MIP-2, etc.), which play various crucial regulatory roles in maintaining the homeostasis of the body’s internal environment [4]. The development of silicosis is closely related to the dynamic cycle of phagocytosis-release-phagocytosis of SiO_2_ particles within alveolar macrophages (AMs). The ingestion of SiO_2_ particles by AMs leads to acute lung inflammation, which is characterized by excessive production of inflammatory mediators and cell death [5]. Subsequently, the ingested SiO_2_ particles are released and reintroduced by other macrophages, thus amplifying the vicious cycle of inflammation and cell death [6]. Depending on the duration of exposure to SiO_2_ particles, silicosis can be subdivided into an acute inflammatory form characterized by silicosis deposition disorder and a chronic form characterized by pulmonary collagen deposition and pulmonary fibrotic remodeling [7,8]. In order to reduce the morbidity and mortality associated with irreversible progressive and incurable silicosis, new therapies are urgently needed to prevent long-term inflammation and collagen deposition in silicosis [1].

Autophagy is a stress-induced response of cells under external stress conditions, which is beneficial to the survival of cells. The entire process, from autophagy induction and phagocytic vesicle formation to the fusion, degradation, and recycling of autophagosomes with lysosomes, is finely and rigorously regulated by autophagy-related genes (ATG) [9]. In the classical bilayer-like autophagosome formation process, the regulation of two independent ubiquitin-like linkage systems (ATG12 and LC3) is required. ATG7, acting as a ubiquitin E1-like activating enzyme, activates both ubiquitin-like proteins ATG12 and LC3, translocating them to the corresponding E2-like enzymes, eventually forming the corresponding ATG12-ATG5 complex, as well as the phosphatidylethanolaminated LC3 (LC3-PE). Studies have shown that conditional knockdown of ATG7 to prevent autophagy will lead to the accumulation of ubiquitinated protein polymers in mouse tissues [10]. At present, it has been found that many functions of macrophages, such as differentiation, polarization, and pathogen elimination, are pivotally regulated by autophagy [11]. In addition, autophagy is closely related to the pathological processes of various diseases, including but not limited to silicosis [12,13,14]. Aberrant autophagic activity has been observed in the lung tissue of a rat silicosis model [15], and silica dust particles have been found in the autophagosomes of patients with silicosis [16]. Mechanism studies have shown that silica dust can cause a cascade stress response in the lysosomes of alveolar macrophages, leading to an increase in autophagosomes, inhibition of autophagy degradation, and promotion of death receptors, mitochondria, and endoplasmic reticulum signaling pathways to mediate the apoptosis of various lung effector cells, thereby promoting the process of pulmonary fibrosis [17]. Lysosome-associated membrane protein 1 (LAMP1) and cathepsin B (CTSB) are important components of lysosomes, which play key roles and functions in cells [18,19]. A study found that the abnormal expression and activity of LAMP1 and CTSB are closely related to the enhancement of the inflammatory response and the formation of fibrosis in the lung tissue of mice exposed to silica dust [20]. Another study pointed out that with the occurrence and development of human silicosis, lysosomes in AMs gradually decreased, while autophagosomes increased. Moreover, the expression of Bcl2-associated Bax and cleaved caspase-3 increased, whereas the expression of Bcl2 decreased [21]. Taken together, the regulation of autophagy may provide a promising new strategy for the prevention and treatment of silicosis.

Tet is a bisbenzylisoquinoline alkaloid extracted from the roots of *Stephamia tetrandra S. Moore* [22]. It is commonly used to treat arthritis, hypertension, tumors, silicosis, liver fibrosis prevention and treatment, liver cell protection, and other conditions in clinical practice [23,24,25]. Both in vitro and in vivo studies have confirmed that Tet is effective in the treatment of silicosis [26,27,28,29]. Currently, tetrandrine (Tet) is the only plant-derived drug approved in China for the treatment of silicosis. Even though a series of studies have shown that Tet exhibits a rich and diverse range of pharmacological effects, such as anti-inflammatory, antioxidant, immunomodulatory, etc. [23,30], effects, its molecular mechanisms of anti-inflammatory and anti-fibrotic action are far from being clarified and are worthy of our high attention.

Existing research has shown that Tet, as a broad-spectrum and effective cell autophagy agonist, can effectively induce the autophagy of various cell lines; and the autophagy is effectively regulated by autophagy inhibitors 3-methyladenine (3-MA) and chloroquine [31]. Another study pointed out that Tet restores TGF-β1-induced autophagy flux impairment while enhancing the interaction between SQSTM1/P62 and MAP1LC3-II [32]. Additionally, our previous research indicated that Tet could target PDPK1 (PDK1), RAC1, KDR, PIK3CA, PTK2, and RPS6KB1 to exert anti-fibrotic effects [33], with further analysis showing that these molecules were closely related to autophagy. To sum up, we speculate that Tet may play an anti-inflammatory and fibrotic role by regulating autophagy in vivo. To our knowledge, there are currently few studies exploring the effect of Tet on the pathological process of silicosis (in vivo) from the perspective of autophagy. Accordingly, the purpose of this study is to explore whether Tet plays a role in alleviating the lung inflammation and fibrosis caused by SiO_2_ by regulating autophagy.

## 2. Materials and Methods

### 2.1. Reagents

Sigma-Aldrich, St. Louis, MO, USA, provided the crystalline silica (Cat# S5631, 80% particle size 1–5 μm, 99% purity). The supplier of tetrandrine was Shanghai Ronghe Pharmaceutical Technology Development Co., Ltd., Shanghai, China (CAS#518-34-3, purity 99.36%). The kits and antibody information utilized in this study are shown in the Supplementary Materials (Table S1).

### 2.2. Experimental Animals and Groups

**Figure 1:**
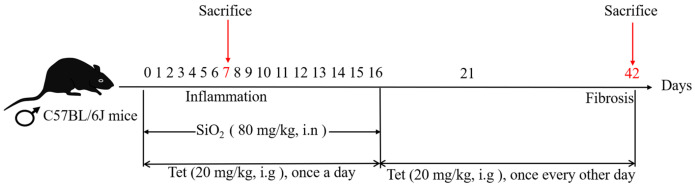
Schematic diagram of experimental plan for early intervention of Tet in silicosis model mice; i.n: intranasal, i.g: intragastrical administration.

Male C57BL/6J mice aged 6 to 8 weeks were raised in a temperature-controlled (22 °C ± 2 °C) environment with a relative humidity of 55% ± 15%, kept in a 12-h light–dark cycle, and given free access to food and water. The mice were provided by Ningxia Medical University Laboratory Animal Center, license number: IACUC-NYLAC-20220219. All procedures were approved by the Medical Ethics Review Committee of Ningxia Medical University (approval No. 2021-N0093). After 7 days of adaptive feeding, mice were subjected to an experiment and divided into 3 treatment groups (*n* = 20) according to the randomization grouping principles as follows: (i) The control group (Ctrl): 0.9% physiological saline (intranasal, i.n) and 90% corn oil + 10% DMSO (intragastrical administration, i.g). (ii) SiO_2_ group: 80 mg/kg SiO_2_ (i.n) and 90% corn oil + 10% DMSO (i.g). (iii) Tet intervention group: 80 mg/kg SiO_2_ (i.n) + 20 mg/kg Tet (i.g). The gavage administration was performed 1 h after nasal drip operation [34], and the dosage of Tet was determined according to pre-experiments conducted by our research group. The establishment of an animal model for silicosis and the pretreatment of SiO_2_ were previously described [35]. The schematic diagram of the exposure and intervention experiments in this study is shown in Figure 1. Body weight was measured and recorded before exposure and intervention operations. The mice were euthanized in batches after isoflurane anesthesia on days 7 and 42, and the lung tissues of mice were taken. Animal experiments shall be strictly carried out in accordance with the operating guidelines for animal experiments.

### 2.3. Organ Coefficient of Lung Tissues

Following the killing and dissection of each group of mice, the intact lungs were collected, the blood on the organ’s surface was washed away with a solution of 0.9% sodium chloride, the connective tissue was removed, then blotted dry with filter paper and weighed, and the organ coefficient of the lungs was determined using the formula “organ coefficient (%) = organ wet weight (g)/body weight (g) × 100.0%”.

### 2.4. Pathological Analysis of Lung Tissues

Three of each group of the upper right lobes of the lung were extracted and fixed for more than 24 h in a paraformaldehyde fixative with a volume fraction of 4%. To determine the level of inflammation and fibrosis in the lung tissue, it was dehydrated, paraffin-embedded, sectioned (5 μm), stained with HE and Masson stain, respectively, with alcohol gradient dehydration, xylene transparent sectioning, neutral resin sealing, and was scanned by a SCIENCE-Digital Section Scanning System. The inflammatory score of HE staining was performed with Szapiel method according to the published literature [36]. Masson trichrome staining was used to observe the degree of pulmonary fibrosis. For statistical analysis, the area of positive expression was calculated by Image J (V1.8.0.112) software.

### 2.5. RT-qRCR

According to the manufacturer’s instructions, total RNA was isolated from lung tissue samples using an RNAsimple Total RNA Kit, and cDNA was synthesized using the FastKing gDNA Dispelling RT SuperMix Kit. The gene expression was determined by RT-qPCR. With the help of the SuperReal PreMix Plus (SYBR Green) Kit, PCR amplification was carried out. A CFX Connect Optics Module fluorescent qPCR apparatus (Bio-Rad, Hercules, CA, USA) was used for data collection and analysis of quantitative PCR tests. In order to normalize the non-PCR-related fluorescence fluctuations between the wells, the internal reference dye signal of *GAPDH* was used to detect each fluorescent reporter signal, three replicate wells were set up for each sample, and the classical 2^−ΔΔCt^ method was used to calculate the relative gene expression [37]. The primer sequences used in the experiment are shown in the Supplementary Materials (Table S2). Primer-BLAST (http://blast.ncbi.nlm.nih.gov, (accessed on 2 October 2022) was used to verify the specificity of all primers before synthesis, and then they were synthesized by Sangon Biotech Co., Ltd., Shanghai, China.

### 2.6. Determination of Hydroxyproline (HYP) Content

An appropriate amount of left lung tissue was taken and its content was determined according to the instructions of the HYP kit. HYP content was expressed in micrograms per gram (μg/g) lung wet weight.

### 2.7. Immunohistochemistry

Dewatering and hydrating paraffin sections of the mouse lung tissue were followed by procedures to repair the antigen, incubated with hydrogen peroxide, and blocked with 5% goat serum at room temperature for 30 min. Primary antibodies against Col-I and Fn were added dropwise and incubated for an overnight period at 4 °C. The secondary antibody labeled with horseradish peroxidase was added, and the nucleus was stained with DAB dye, then re-stained with hematoxylin dye, and finally dehydrated. The SCIENCE digital section digitalization system was employed for slice scanning.

### 2.8. Western Blot (WB)

The right lobes of the lung tissue were removed and immediately frozen in liquid nitrogen and stored at −80 °C in an ultra-low temperature freezer. An appropriate amount of lung tissue was taken and added with a commonly used tissue lysis buffer (including phosphatase inhibitor, phenylmethylsulfonyl fluoride, and protease inhibitor). The tissue was mechanically ground and frozen for 10 min to extract the total protein. The protein concentration was determined using the BCA protein assay kit. The protein concentration in all samples was normalized, and SDS-PAGE electrophoresis was performed. After electrophoresis, the protein was transferred onto a polyvinylidene fluoride membrane. The membrane-carrying protein was blocked by 5% non-fat milk at room temperature for 2 h. After overnight incubation at 4 °C with the primary antibody, the membrane was washed with TBST buffer, incubated with the corresponding secondary antibody at a 1:5000 dilution ratio at room temperature for 2 h, and washed with TBST buffer. The membrane was placed in an electrochemiluminescence chromogenic solution and captured by a gel imager. The optical density of the bands was analyzed by Image J software. The relative protein expression was calculated according to the ratio of the target protein to the internal reference protein.

### 2.9. Immunofluorescence

Dewaxed and hydrated mouse lung tissue paraffin slices were rinsed with PBS, permeabilized with 0.25% Triton X-100 for 10 min, and blocked with 5% goat serum at room temperature for 30 min. The samples were incubated overnight with primary antibodies ATG7, LC3B, and P62 at 4 °C, and then incubated with the secondary antibody. After washing, the samples were incubated in a wet box at room temperature and away from light for 2 h. After washing, the sealing agent containing DAPI was added dropwise in a dark place, and the cover glass was sealed. Images were observed and collected under a fluorescence microscope (DM2500, Leica, Wetzlar, Germany), and the images were semi-quantitatively analyzed using Image J.

### 2.10. Transmission Electron Microscopy (TEM)

All instruments, wax plates, and fixatives were pre-cooled in advance. After the mice were sacrificed, the lung tissue was immediately washed with 0.1 mol/L phosphate buffer solution (pH 7.4), and the right septal lung tissue was cut into 1 mm^3^ small pieces. During the operation, the impact of mechanical damage (such as traction, contusion, and compression) on the lung tissue structure should be reduced. Then, the tissue slices were placed on a wax plate with glutaraldehyde fixative (within 5 s). The air in the lung tissue was expelled by gently pressing the tissue with a pre-cooled glass slide until the small bubbles disappeared. Subsequently, the lung tissue was fully shaken in the fixative to completely sink and was fixed for 24 h. Finally, the transmission electron microscopy (TEM) examination was conducted by professional technicians from the Science and Technology Center of Ningxia Medical University.

### 2.11. Statistical Analysis

Statistical analysis and plotting were conducted using SPSS 26.0 and Prism 8.0 software, respectively. The measurement data conforming to normal distribution through normality testing were reported as the mean standard deviation (mean ± SD). The *t*-test was used to analyze differences between two groups, and the one-way ANOVA was used to compare means across multiple groups. When the homogeneity of variance was satisfied, the LSD-*t* test was employed for post hoc multiple comparisons; conversely, Dunnett’s *t*-test was utilized. For *p* < 0.05, the differences were considered to be statistically significant.

## 3. Results

### 3.1. Tet Could Counteract the Adverse Effects of SiO_2_ Exposure on Body Weight and Lung Organ Coefficient of Mice to a Certain Extent

**Figure 2:**
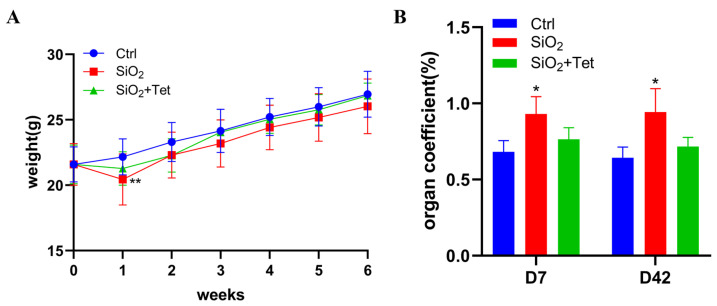
Changes in body weight and pulmonary organ coefficients of mice. (**A**) Changes in body weight of mice in each group at different time points (*n =* 8). (**B**) Lung organ coefficients of mice at 7 and 42 days (*n =* 6). Results are expressed as mean ± SD, compared with the control group, * *p* < 0.05. ** *p* < 0.01.

After one week of exposure, the weight of mice in the SiO_2_ group decreased significantly compared with that in the control group (*p* < 0.05). After that, the mice’s body weight gradually grew in each group (Figure 2A). On the 7th and 42nd day of SiO_2_ exposure, compared with the control group, the lung organ coefficient of mice in SiO_2_ group increased significantly (*p* < 0.05), which might be due to the combined impact of increased collagen content and aggravated pulmonary edema caused by SiO_2_ exposure. Compared with the SiO_2_ group, the lung organ coefficient of mice in the Tet intervention group decreased, although the difference was not statistically significant (Figure 2B).

### 3.2. Tet Could Effectively Alleviate Pulmonary Inflammation and Fibrosis Caused by SiO_2_ in Mice

#### 3.2.1. Hematoxylin-Eosin (HE) Staining

**Figure 3:**
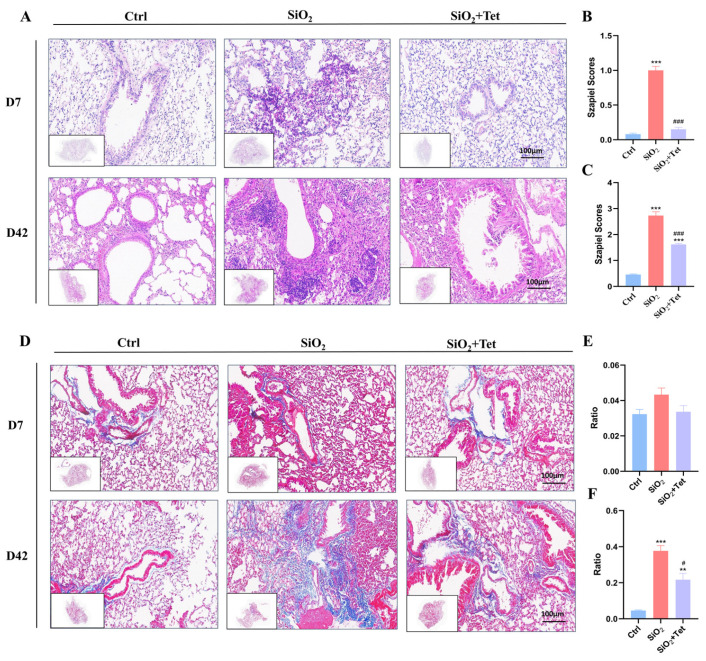
Histopathological changes in mouse lungs. (**A**) Representative HE staining images of lung sections (200×) from each group of mice on days 7 and 42 of SiO_2_ exposure (*n =* 3). (**B**) Szapiel scores for HE staining after the 7-day experiment were statistically analyzed. (**C**) The statistical evaluation of the Szapiel scores for the 42-day experiment’s HE staining. (**D**) The representative images of Masson staining of lung tissue sections from each group of mice (200×) (*n =* 3). (**E**) The results of the quantitative Masson staining analysis after the 7-day trial. (**F**) The outcomes of the quantitative Masson staining analysis after the 42-day experiment. Results are expressed as mean ± SD, compared with the control group, *** *p* < 0.001, ** *p* < 0.01; Compared with SiO_2_ group, ^#^ *p* < 0.05, ^###^ *p* < 0.001.

The results of HE staining (Figure 3A) demonstrated a clear alveolar structure and thin alveolar walls in the lung tissues of the control group mice, whereas the SiO_2_ group’s lung tissue revealed obvious inflammatory cell infiltration, thickened alveolar walls, and widened and fused interstitial characteristics. Compared to the experimental results on day 7, the histopathological changes were more severe on day 42, mainly manifested as macrophage aggregation, fibrous tissue hyperplasia, and parenchymalization of the lung mass. In comparison to the SiO_2_ group, the degree of alveolitis in the lung tissue of the Tet intervention group mice was relatively mild on days 7 and 42. The same outcomes were observed using the Szapiel inflammatory score (Figure 3B,C).

#### 3.2.2. Masson Staining

Masson staining results (Figure 3D) showed that on day 7, the alveolar structure of the lung tissue in the control group mice was intact, and no obvious collagen fiber deposition was found, with only a small amount of stromal collagen fibers (blue stained area) present around the walls of the large airways and blood vessels. The Masson staining results of lung tissues in the SiO_2_ group and Tet intervention group mice were similar to those in the control group, which may be related to the fact that inflammation is mainly present in the early stage of silica dust exposure, while tissue fibrosis repair mainly occurs in the mid to late stage. The quantitative analysis of collagen fibers stained by Masson showed that there was no significant difference in collagen fiber area between the SiO_2_ group and the Tet intervention group in the lung tissues of mice compared to the control group (Figure 3E).

On day 42, blue-stained collagen fiber deposition could be observed in the alveolar septa and bronchial walls of SiO_2_ group mice, and the degree of collagen deposition in the Tet intervention group was lower than that in the SiO_2_ group. Similarly, quantitative analysis results indicate that the area of collagen fibers in the lung tissues of SiO_2_ group mice was significantly increased compared to the control group (*p* < 0.05). Compared with the SiO_2_ group, the area of collagen fibers in the Tet intervention group decreased significantly (*p* < 0.05) (Figure 3F).

### 3.3. Tet Could Relieve SiO_2_-Induced Inflammation by Inhibiting Inflammatory Cytokines TNF-α, IL-1β, MCP-1, and TGF-β1 mRNA in the Lung Tissues of Mice

**Figure 4:**
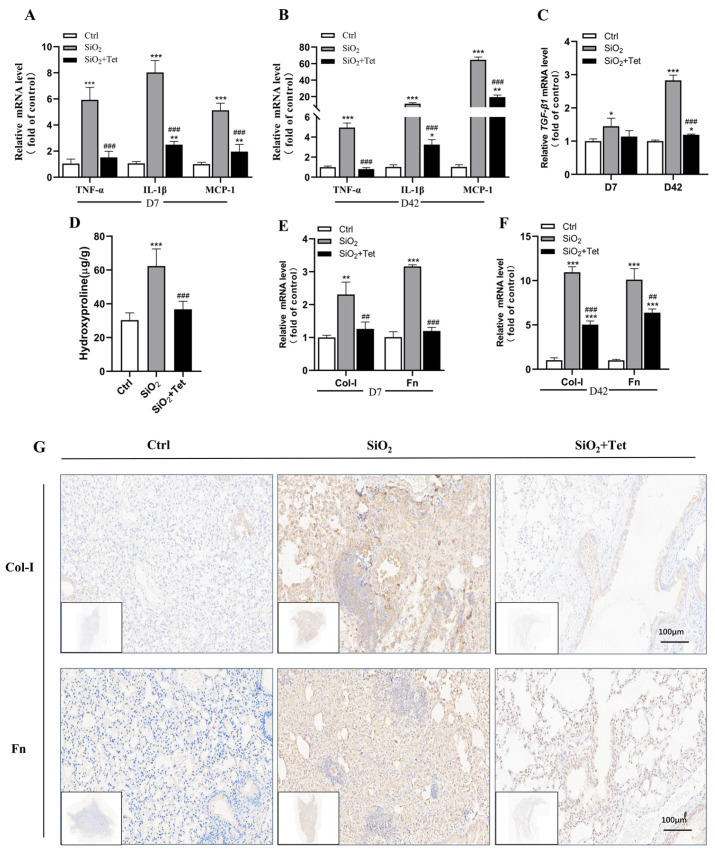
Effect of Tet intervention on the expression of inflammatory and fibrotic markers in the lung tissues of SiO_2_-exposed mice. (**A**) The levels of *TNF-α*, *IL-1β*, and *MCP-1* mRNA expression in mouse lung tissues after the 7-day experiment. (*n =* 6). (**B**) *TNF-α*, *IL-1β*, and *MCP-1* mRNA expression levels in mouse lung tissues at the end of the 42-day experiment (*n =* 6). (**C**) *TGF-β1* mRNA expression levels in mouse lung tissues at the conclusion of the 7-day and 42-day experiments (*n =* 3). (**D**) Analysis of HYP levels in mouse lung tissues after a 42-day experiment (*n =* 6). (**E**) *Col-I* and *Fn* mRNA expression levels in mouse lung tissues after a 7-day experiment (*n =* 3). (**F**) *Col-I* and Fn mRNA expression levels in mouse lung tissues after a 42-day experiment. (*n =* 3). (**G**) IF results of lung tissue Col-I and Fn in mice at the end of the 42-day experiment (200×) (*n =* 3). Results are expressed as mean ± SD, compared with the control group, * *p* < 0.05, ** *p* < 0.01, *** *p* < 0.001; Compared with SiO_2_ group, ^##^ *p* < 0.01, ^###^ *p* < 0.001.

According to the results of RT-qPCR, on day 7, SiO_2_ exposure increased the levels of *TNF-α*, *IL-1β*, *MCP-1*, and *TGF-β1* mRNA expression in the lung tissue of mice by 4.88, 6.97, 4.12, and 0.45 times, respectively, in comparison to the control group (all *p* < 0.05). In addition, compared with the SiO_2_ group, the expression levels of *TNF-α*, *IL-1β*, *MCP-1*, and *TGF-β1* mRNA in the Tet intervention group decreased by 0.74, 0.69, 0.62, and 0.22 times, respectively (all *p* < 0.05) (Figure 4A,C).

On day 42, SiO_2_ exposure upregulated *TNF-α*, *IL-1β*, *MCP-1*, and *TGF-β1* mRNA expression levels in the lung tissues of mice by 3.94, 10.23, 63.59, and 1.83-fold, respectively, compared with the control group (all *p* < 0.05). By comparing the expression levels of inflammatory factors on the 7th and 42nd days, it was discovered that, except *TNF-α*, the enhanced expression of *IL-1β*, *MCP-1*, and *TGF-β1* mRNA on day 42 after SiO_2_ exposure were more significant than those on day 7. In the Tet intervention group compared to the SiO_2_ group, the expression *TNF-α*, *IL-1β*, *MCP-1*, and *TGF-β1* mRNA was downregulated by 0.84, 0.71, 0.70, and 0.57 times, respectively (all *p* < 0.05) (Figure 4B,C).

### 3.4. Tet Could Alleviate SiO_2_-Induced Fibrosis by Inhibiting the Expression of Markers of Positive Regulation of Fibrosis—HYP, Col-I, and Fn in Lung Tissue

#### 3.4.1. Effect of Tet Intervention on the HYP Concentration in the Lung Tissues of Mice after 42 Days of SiO_2_ Exposure

Based on the HYP assay results, mice in the SiO_2_ group had a 105.57% (*p* < 0.05) higher HYP content in their lung tissue compared to mice in the control group. In addition, the concentration of HYP in the lung tissues of mice in the Tet intervention group declined by 41.03% (*p* < 0.05) relative to the SiO_2_ group (Figure 4D).

#### 3.4.2. Effect of Tet Intervention on Col-I and Fn in the Lung Tissues of Mice Exposed to SiO_2_

Results from RT-qPCR revealed that compared with the control group, the contents of *Col-I* and *Fn* mRNA in the lung tissues of mice exposed to SiO_2_ increased by 1.31 and 2.15 times (all *p* < 0.05), respectively, on the 7th day, while the contents of Col-I and Fn in the lung tissues of mice treated with Tet decreased by 0.45 and 0.62 times (all *p* < 0.05) compared with SiO_2_ group (Figure 4E).

The expression levels of *Col-I* and *Fn* mRNA in the lung tissues of mice exposed to SiO_2_ on the 42nd day were higher than that of the control group on the 7th day, and increased by 9.92 and 9.10 times, respectively. In the Tet intervention group, the expression of *Col-I* and *Fn* mRNA were significantly decreased by 0.54 and 0.37-fold, respectively compared to the SiO_2_ group (all *p* < 0.05) (Figure 4F).

In agreement with the RT-qPCR results, the IHC analysis of the lung tissues from SiO_2_-exposed mice on day 42 revealed that the expression of Col-I and Fn protein were significantly higher compared to the control group and significantly lower in the Tet intervention group compared to the SiO_2_ group (Figure 4G).

### 3.5. Tet Could Regulate the Expression of Key Molecules ATG7, LC3B, and P62 in the Autophagy Pathway Induced by Silica in Mouse Lung Tissue

#### 3.5.1. Tet Intervention Increases ATG7 Expression in the Lung Tissues of Mice Exposed to SiO_2_

**Figure 5:**
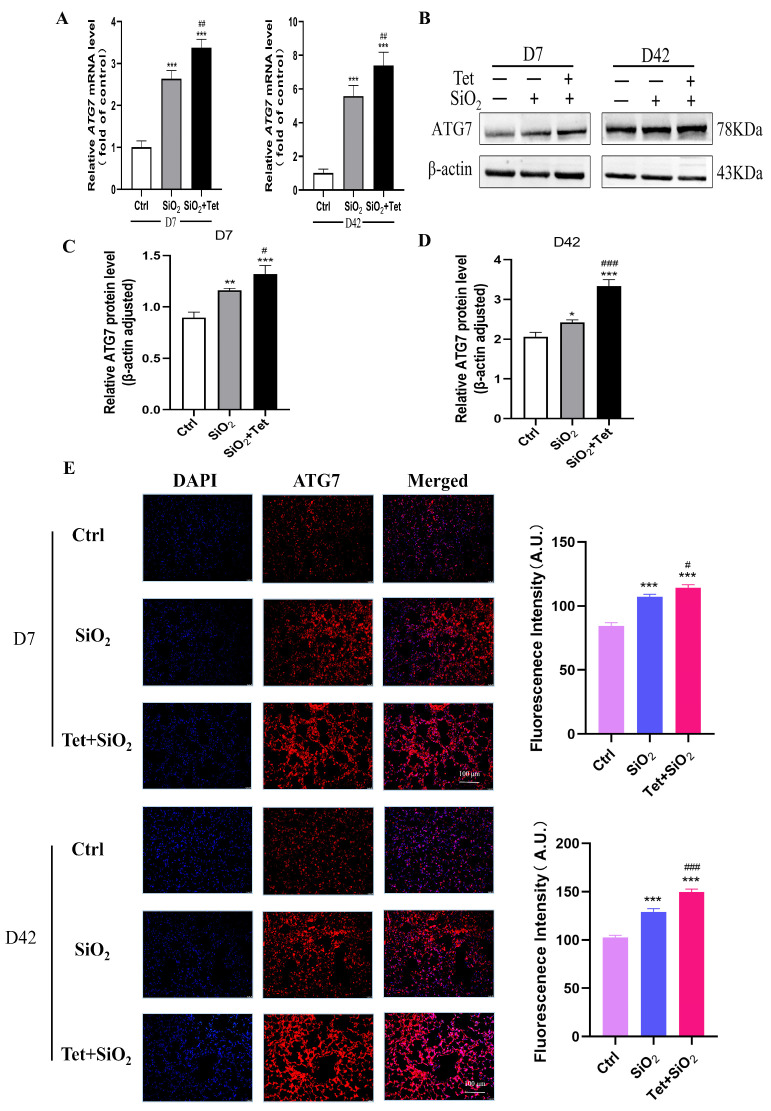
Tet promotes the expression of ATG7 in the lung tissues of mice exposed to SiO_2_. (**A**) *ATG7* mRNA levels were monitored by RT-qPCR in the lung tissues of mice exposed to SiO_2_ at 7 and 42 days (*n =* 3). (**B**–**D**) WB band diagram and quantitative analysis results of ATG7 protein in the lung tissue of mice exposed to SiO_2_ at 7 and 42 days. (**E**) IF analysis of the distribution and expression of ATG7 in the lung tissues of mice exposed to SiO_2_ at 7 and 42 days (200×) (*n =* 3). Results are expressed as mean ± SD, compared with the control group, * *p* < 0.05, ** *p* < 0.01, *** *p* < 0.001; Compared with SiO_2_ group, ^#^ *p* < 0.05, ^##^ *p* < 0.01, ^###^ *p* < 0.001.

ATG7, as a crucial component of the autophagy genesis pathway, plays a key role in the development of traditional autophagosomes with a bilayer-like structure. So, in order to investigate the role of ATG7 in SiO_2_-induced inflammation and fibrosis, RT-qPCR and IF were used to detect the changes of ATG7 at different time points of SiO_2_ exposure. According to the results of RT-qPCR, it could be seen that the SiO_2_ group considerably increased the expression of *ATG7* mRNA when compared to the control group (*p* < 0.05), and the Tet intervention group further increased the expression of *ATG7* mRNA when compared to SiO_2_ group (*p* < 0.05) (Figure 5A).

In order to further clarify the expression of ATG7 in lung tissue, we detected the expression and distribution of ATG7 in lung tissue by WB and IF. WB results showed that in the lung tissue of silicosis model mice, compared with the control group, SiO_2_ significantly increased the expression of the ATG7 protein in lung tissue; compared with the SiO_2_ group, Tet intervention further promoted the expression of ATG7 (Figure 5B–D). IF also found that Tet could effectively promote the expression of ATG7 in the cytoplasm (Figure 5E).

#### 3.5.2. Tet Intervention Downregulated the Expression of LC3B in the Lung Tissues of Mice Exposed to SiO_2_

**Figure 6:**
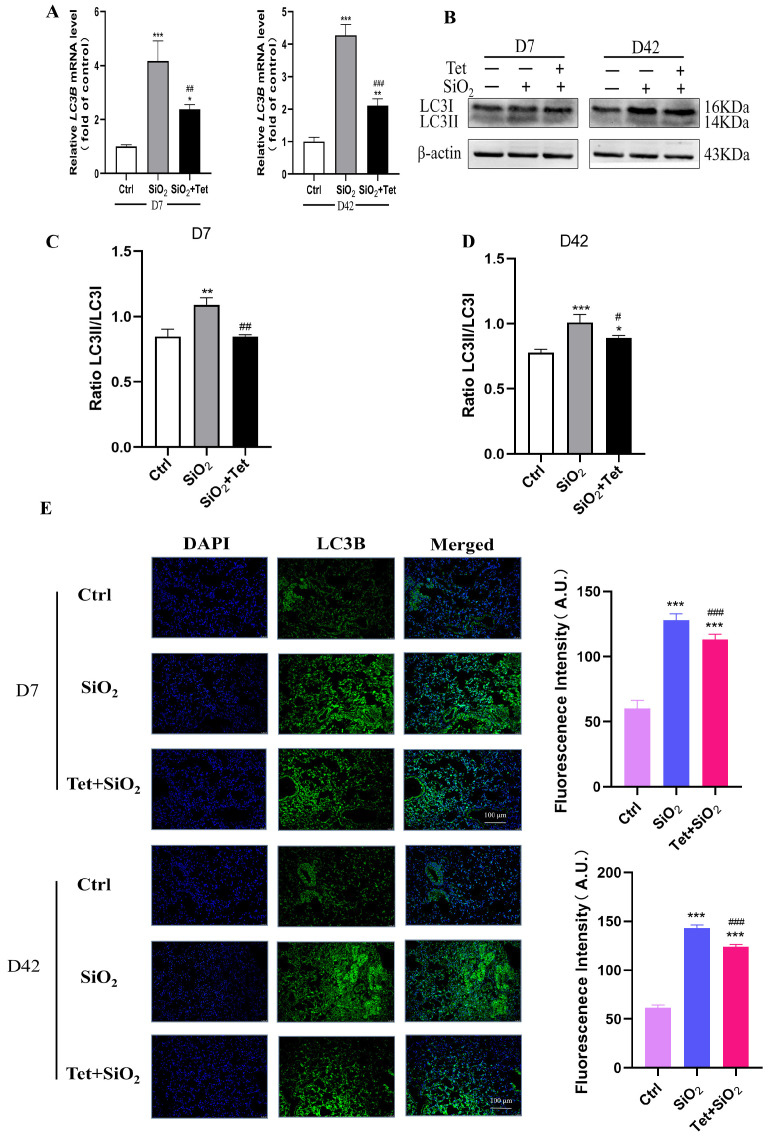
Tet treatment reduces LC3B expression in the lung tissues of mice exposed to SiO_2_. (**A**) *LC3B* mRNA alterations in the lung tissues of mice at 7 and 42 days were detected using RT-qPCR (*n =* 3). (**B**–**D**) WB band diagram and quantitative analysis results of LC3 protein in the lung tissue of mice after 7 and 42 days of silica exposure. (**E**) LC3B expression alterations in the lung tissues of mice that had been exposed to SiO_2_ for 7 and 42 days were detected using IF (200×) (*n =* 3). Results are expressed as mean ± SD, compared with the control group, * *p* < 0.05, ** *p* < 0.01, *** *p* < 0.001; Compared with SiO_2_ group, ^#^ *p* < 0.05, ^##^ *p* < 0.01, ^###^ *p* < 0.001.

LC3B is a microtubule-associated protein, which is one of the markers of the autophagy pathway, and its expression level can be used to evaluate the degree of autophagy activity. Results from RT-qPCR revealed that, in comparison to the control group, *LC3B* mRNA expression levels were considerably higher on days 7 and 42 of SiO_2_ exposure (*p* < 0.05). Compared with the SiO_2_ group, *LC3B* mRNA in the Tet intervention group decreased but was still higher than that in the control group (*p* < 0.05) (Figure 6A). In order to further clarify the situation of autophagic flux, we used WB to detect the change of the LC3 protein from cytoplasmic LC3I to membrane LC3II. The results showed that compared with the control group, the protein expression of autophagy-related protein LC3II was increased in the SiO_2_ group. Compared with the SiO_2_ group, the expression of LC3II in the Tet intervention group was significantly decreased (Figure 6B–D). The results of immunofluorescence experiments showed that LC3B was mainly localized in the cytoplasm, and its expression was consistent with the results of RT-qPCR and WB (Figure 6E).

#### 3.5.3. Tet Intervention Decreased the Expression of P62 in the Lung Tissues of Mice Exposed to SiO_2_

**Figure 7:**
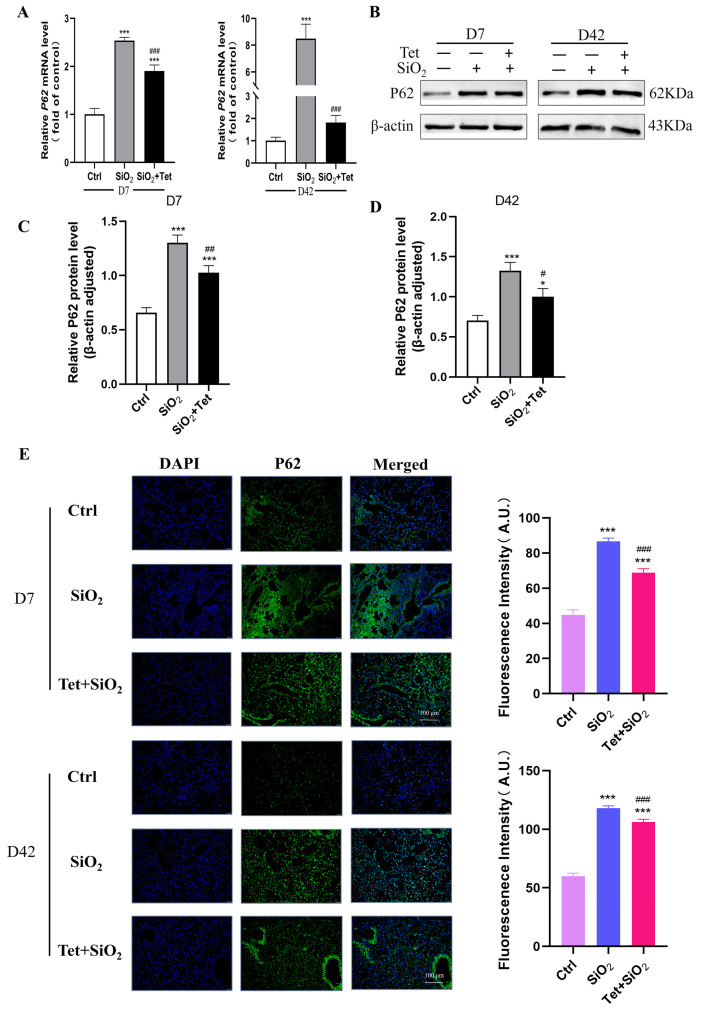
Tet intervention downregulates P62 expression in the lung tissues of mice exposed to SiO_2_. (**A**) Changes in *P62* mRNA were found using RT-qPCR in the lung tissues of mice that had been exposed to SiO_2_ for 7 and 42 days (*n =* 3). (**B**–**D**) WB band diagram and quantitative analysis results of P62 protein in the lung tissue of mice after 7 and 42 days of silica exposure. (**E**) P62 protein expression alterations in the lung tissues of mice exposed to SiO_2_ for 7 and 42 days were detected using IF. (200×) (*n =* 3). Results are expressed as mean ± SD, compared with the control group, * *p* < 0.05, *** *p* < 0.001; Compared with SiO_2_ group, ^#^ *p* < 0.05, ^##^ *p* < 0.01, ^###^ *p* < 0.001.

P62 is a protein that is an autophagic substrate and binds to LC3, which helps autophagosomes form and degrade. The results of RT-qPCR showed that the expression levels of *P62* mRNA were significantly higher on the 7th and 42nd day of SiO_2_ exposure than those in the control group. When compared to SiO_2_ group, the Tet intervention group’s *P62* mRNA expression level was considerably lower (all *p* < 0.05) (Figure 7A). The results of WB showed that the expression of P62 protein in the lung tissue of silicosis model mice was significantly increased by SiO_2_ compared with Ctrl group at 7 days and 56 days. Compared with the SiO_2_ group, Tet intervention effectively reduced the deposition of P62 protein, and the difference was statistically significant (Figure 7B–D). In addition, IF detection also found that Tet could effectively reduce the deposition of P62 protein in mouse lung tissue (Figure 7E).

### 3.6. Tet Promotes the Recovery of Silica-Induced Autophagic Lysosomal System Function

#### 3.6.1. Tet Could Affect the Expression of LAMP1 and CTSB Protein in the Lung Tissue of SiO_2_-Exposed Mice

**Figure 8:**
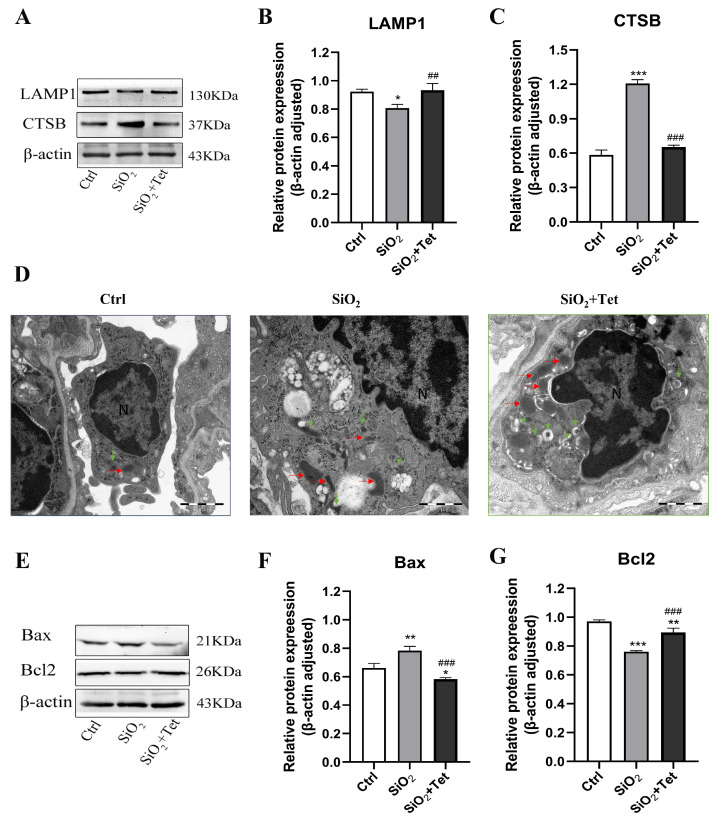
Effects of Tet intervention on the expression of lysosomal function indexes and apoptotic factors in lung tissue of SiO_2_ exposed mice. (**A**–**C**) WB band pattern and quantitative analysis results of LAMP1 and CTSB protein in lung tissue of mice in each group after 42 days of silica exposure. (**D**) The ultrastructure of alveolar macrophages in each group after 42 days of silica exposure observed by Transmission electron microscopy (N represents nucleus. Red horizontal arrow represents lysosome. Green vertical arrow represents autophagic lysosome). The scale in Figure is 1 μm, and the magnification is 30,000 times. (**E**–**G**) WB band diagram and quantitative analysis results of Bax and Bcl2 protein in lung tissue of mice in each group after 42 days of silica exposure. Results are expressed as mean ± SD (*n =* 3). Compared with the control group: * *p* < 0.05, ** *p* < 0.01, *** *p* < 0.001. Compared with SiO_2_ group: ^##^ *p* < 0.01, ^###^ *p* < 0.001.

As shown in Figure 8A–C, compared with the control group, the relative protein expression level of LAMP1 in the lung tissue of mice decreased after 42 days of silica exposure, while the relative protein expression level of CTSB increased. Tet could reverse the decrease of LAMP1 and the increase of CTSB caused by silica exposure (*p* < 0.05). This suggests that Tet may improve lysosomal function by regulating the expression of LAMP1 and CTSB in the lung tissue of silica-exposed mice.

#### 3.6.2. Tet Could Affect the Formation of Autophagosomes and Lysosomes in the Lung Tissue of SiO_2_-Exposed Mice

The results of TEM of lung tissue showed that, in the control group, there were scattered lysosomes and occasional autophagic lysosomes. The membrane coating was normal, and no obvious abnormalities were found in the cytoplasm and other organelles. In the SiO_2_ model group, swollen mitochondria and swollen and vacuolated autophagic lysosomes were observed. Compared with the SiO_2_ model group, more morphologically normal lysosomes and autophagosomes could be observed in the Tet intervention group (Figure 8D).

### 3.7. Tet Improved SiO_2_-Induced Apoptosis by Regulating the Release of Apoptotic Factors Bax and Bcl2 in Lung Tissue

The results of WB showed that, compared with the control group, the relative expression level of Bax protein in the lung tissue of SiO_2_-exposed mice increased, while the level of Bcl2 protein decreased (*p* < 0.05). Compared with the SiO_2_ group, the relative expression level of Bax protein in the lung tissue of the Tet intervention group was significantly decreased, while the level of Bcl2 protein was significantly increased (*p* < 0.05) (Figure 8E–G).

## 4. Discussion

Silicosis is a chronic inflammatory reaction and progressive fibrosis caused by repeated exposure to inhalable silica dust particles, which eventually leads to extracellular matrix deposition and damage to the alveolar structure. Due to its high morbidity and mortality, silicosis has become a global public health problem [2]. However, because of its complex pathogenesis, difficulty in early diagnosis, and poor prognosis, there is still a lack of safe and effective treatment in clinical practice. Therefore, with the goal of delaying the development of pulmonary fibrosis, exploring the molecular mechanisms of silicosis pathogenesis and finding potential therapeutic targets undoubtedly have important practical significance. Presently, the experimental studies on exploring its molecular mechanism and seeking potential therapeutic targets are mainly carried out by establishing animal models. The existing methods of establishing silicosis animal models mainly include intratracheal perfusion, dynamic nebulization inhalation, and intranasal drip, among which, intratracheal perfusion is a commonly used method. However, it is an invasive method, which may cause mechanical damage to the inner wall of mouse trachea [38]. At the same time, it is difficult for this method to simulate the real exposure scenarios of the occupational population. Considering the advantages of simple operation, good repeatability, and non-invasiveness, an intranasal drip was employed as the exposure pathway for SiO_2_ in this study. The experimental details of modeling were referenced from the published literature [35].

AMs are the “sentinels” against exogenous harmful factors. They are also the main target cells of silica dust. By repeatedly and continuously stimulating AMs and other cells to secrete a great quantity of cytokines, growth factors, and oxidants, silica dust particles promote the proliferation of fibrotic cells and the production of extracellular matrix, thus resulting in fibrosis [39]. A large number of research has shown that macrophage autophagy is closely related to the occurrence and development of fibrotic diseases such as silicosis [11,17]. Silica dust can stress the lysosomes of alveolar macrophages, lead to the increase of autophagy and the inhibition of autophagy degradation, and promote the apoptosis of various pulmonary effector cells mediated by death receptor, mitochondria, and endoplasmic reticulum signaling pathways, thus promoting the process of pulmonary fibrosis [14]. Relevant studies have demonstrated that, compared with wild-type Lyz2-cre mice exposed to crystalline silica (CS), the lung tissue of ATG7 (flox/flox) Lyz2-cre mice exposed to CS showed a more significant increase in macrophage-led inflammatory infiltrates and pro-inflammatory factors. Vitamin D could reduce lung injury by inducing autophagy to restore anti-inflammatory M2 type macrophages [40]. According to the research of Xie et al. [12], after exposure to CS, the expression levels of autophagy markers LC-II/LC3-I, P62, Beclin1, and growth arrest specific protein 6 (Gas6) in RAW264.7 macrophages increased, and the secretion of inflammatory cytokines decreased. Further studies found that autophagy inhibitors, such as 3-MA, could promote the release of inflammatory cytokines. The above information suggests that autophagy dysfunction is an important step in the progress of silicosis, and the treatment by regulating autophagy activity may become an effective therapy to intervene in silicosis.

Tet is the main extract of Chinese medicine *Stephania tetrandra S. Moore*, which has been used in the clinical treatment of silicosis for 50 years. Clinical studies have found that Tet can control and postpone the pathological process of pulmonary fibrosis in silicosis patients (chest HRCT imaging evidence, lung function, changes of molecular indexes related to lung inflammation and fibrosis, etc.) [26,27,29]. In this study, the results of histopathological experiments showed that Tet intervention could significantly alleviate lung inflammation and fibrosis in mice caused by SiO_2_. The biochemical test results indicated that Tet intervention could significantly downregulate the secretion of inflammatory and fibrotic factors (TNF-α, IL-1β, MCP-1, and TGF-β1) in mouse lung tissues, as well as the deposition of fibrotic related proteins (HYP, Col-I, and Fn).

On the basis of a comprehensive analysis of the literature research results [31,41,42,43] and our previous work [33], we speculate that Tet may mitigate lung inflammation and fibrosis induced by SiO_2_ in mice by regulating macrophage autophagy. Based on the related studies, ATG7, LC3B, and P62, which play important roles in autophagy regulation, were selected as the research focus, and systematically studied the effects of SiO_2_ exposure and/or Tet intervention on these indexes, with a view to reveal whether Tet intervention may relieve lung inflammation and fibrosis induced by SiO_2_ through the autophagy pathway.

ATG7 is an important gene in autophagy, and the protein encoded by it is a key enzyme in autophagosome formation. It can catalyze the binding of ATG12 and ATG5 to form the ATG12-ATG5 complex, and then participate in the phosphorylation of ATG8/LC3 to promote the formation and maturation of autophagosomes. Additionally, ATG7 also participates in biological processes such as protein degradation in the cytoplasm, mitochondrial quality control, and immune regulation. At present, researchers have deeply explored the roles and mechanisms of ATG7 in autophagy through gene knockout, gene expression regulation, protein interaction, and other methods. Related studies have shown that the deletion of ATG7 will hinder the autophagy process, thus affecting the metabolism and survival of cells. Activating ATG7 can effectively delay the pathogenesis of various diseases, including but not limited to neurodegenerative diseases, cardiovascular diseases, and lysosomal storage diseases [44]. This study found that SiO_2_ exposure significantly upregulated the expression level of ATG7 in mouse lung tissues (gene and protein level), consistent with the research results of Komatsu et al. [44]. It is worth noting that Tet intervention could further upregulate the expression of ATG7 in mouse lung tissues (gene and protein level).

LC3B is a key protein in autophagy, which is involved in the formation, extension, and closure of autophagy. The main function of LC3B is to act as a marker protein of autophagosome membrane. It binds to phosphatidylethanolamine (PE) on the outer part of the autophagosome membrane to form LC3B-II, which can bind to the autophagosome membrane to promote the formation and extension of the autophagosome, and finally transports the degraded organelles or proteins to lysosomes for degradation. In the study of AMs isolated from silicosis patients, it was found that autophagy-related proteins increased, suggesting that autophagy is involved in the pathological process of silicosis fibrosis [21]. Similar results were also found in the lung tissues and AMs in silicosis model mice [20,45]. Notably, in this study, it was found that the expression levels of LC3B mRNA and protein in the SiO_2_ group were significantly higher than those in the control group. Tet intervention could downregulate the expression of LC3B in the lung tissues of SiO_2_-exposed mice, suggesting that Tet intervention might relieve the damage effects of autophagy degradation obstruction, autophagy accumulation, and lysosome destruction caused by SiO_2_ exposure.

P62 is the receptor protein of abnormal misfolding proteins and organelles that need to be degraded. It can interact with ubiquitin through the hydroxyl terminal of the UBA region and self-assembles with the amino terminal of the PB1 region, thus forming large protein aggregates containing ubiquitinated proteins. Based on the above information, it can be easily seen that P62 protein is an important autophagy-related protein, which can reflect the patency of autophagy flow. Generally speaking, its reduction indicates that the process of protein degradation through autophagy is completed. Contrarily, its increase indicates that the degradation of misfolded proteins is hindered [46,47]; that is, the autophagy flow is not completed. In this study, on one hand, SiO_2_ exposure could significantly increase the expression level of P62 in mouse lung tissues (gene and protein level), suggesting that SiO_2_ exposure could lead to the inhibition of the autophagy degradation process, thereby preventing the degradation of P62 protein. On the other hand, Tet intervention could antagonize the upregulation of P62 induced by SiO_2_ to a certain extent (gene and protein level), suggesting that Tet could effectively reduce the deposition of P62 protein in the lung tissue of mice.

In addition to what has already been discussed above, we should also note that the results of TEM experiments also intuitively confirm the clear role of macrophage-dominated autophagy in the relief of silica-induced pulmonary inflammation and fibrosis by Tet, and the WB experimental results suggest that the apoptosis signaling pathways related to autophagy may also play a certain role in it.

## 5. Conclusions

Based on the existing research evidence, SiO_2_ can induce inflammation and fibrosis in the lung tissue of mice, promote the occurrence of autophagy, but cause the blockage of autophagy flow. Tet, a natural plant drug that has been widely used in clinical practice, can alleviate silica-induced lung inflammation and fibrosis, which may be achieved by regulating the expression of key molecules in the autophagy process and associated apoptotic pathway. Next, we will combine in vivo and in vitro toxicological experimental methods to explore the molecular mechanisms of Tet in alleviating pulmonary inflammation and fibrosis caused by silica from the perspective of autophagy.

## Data Availability

All data generated or analyzed during this study are included in this published article.

## Supplementary Materials

The following supporting information can be downloaded at: https://www.mdpi.com/article/10.3390/toxics11090765/s1. Table S1. Detailed information about the kits and antibodies used in this study; Table S2. Primer sequence information used in this study.
