# Supplementary material for: Preliminary Study on the Effect and Molecular Mechanism of Tetrandrine in Alleviating Pulmonary Inflammation and Fibrosis Induced by Silicon Dioxide
Source: Toxics. 2023 Sep 9;11(9):765. doi: 10.3390/toxics11090765 (PMC10536946; doi:10.3390/toxics11090765)
Supplement: Supplementary file 1 [file toxics-11-00765-s001.zip › toxics-2576449-supplementary.pdf]

## Supplementary Materials

**Table S1.** Detailed information about the kits and antibodies used in this study.

| Kits / Antibodies | Manufacturers | Cat No. | Application (dilution ratio) |
|---|---|---|---|
| RNAsimple Total RNA Kit | TIANGEN Biotech (Beijing) Co., Ltd., China | DP419 | - |
| FastKing gDNA Dispelling RT SuperMix Kit | | KR118 | - |
| RealUniversal Color PreMix (SYBR Green) Kit | | FP205 | - |
| Hydroxyproline (HYP) Content Assay Kit | Beijing Solarbio Science & Technology Co., Ltd., China | BC0255 | - |
| Rabbit polyclonal anti-Collagen I (Col-I) antibody | Affinity Biosciences (Jiangsu), China | AF7001 | IHC (1:200) |
| Rabbit polyclonal anti-Fibronectin (Fn) antibody | | AF5335 | IHC (1:200) |
| Rabbit monoclonal anti-LC3B antibody | Abcam | AB192890 | WB (1:4000) |
| Rabbit monoclonal anti-p62/SQSTM1 Antibody | Abmart Inc. China | T55546 | WB (1:5000) |
| Rabbit polyclonal anti-ATG7 antibody | Affinity Biosciences (Jiangsu), China | DF6130 | IF (1:200) |
| Rabbit polyclonal anti-LC3B antibody | | AF4650 | IF (1:200) |
| Rabbit polyclonal anti-SQSTM1/P62 antibody | | AF5384 | IF (1:200) |
| Rabbit polyclonal anti-β-actin antibody | | AF7018 | WB (1:5000) |
| Rabbit polyclonal anti-LAMP1 antibody | | DF4806 | WB (1:1000) |
| Rabbit polyclonal anti-CTSB antibody | | AF5189 | WB (1:500) |
| Rabbit polyclonal anti-Bax antibody | | AF0120 | WB (1:1000) |
| Rabbit polyclonal anti-Bcl2 antibody | | AF6139 | WB (1:1000) |
| Rhodamine-labeled goat anti-rat IgG (H+L) (affinity purified) | Beijing Zhongshan Jinqiao Biotechnology Co., Ltd., China | ZF-0318 | IF (1:100) |
| Alexa Fluor® 488-labeled goat anti-rabbit IgG (H+L) (affinity purified) | | ZF-0511 | IF (1:400) |
| Rabbit Two-Step Assay Kit (Rabbit Enhanced Polymer Assay System) | | PV-9001 | IHC |
| Horseradish peroxidase-labeled goat anti-rabbit IgG (H + L) | Affinity Biosciences (Jiangsu), China | S0001 | WB（1:5000） |

**Table S2.** Primer sequence information used in this study.

| Gene | GenBank Accession No. | Forward primer (5'-3') | Reverse primer (5'-3') | Fragment Length |
|---|---|---|---|---|
| *GAPDH* | NM_001411843 | GTATTGGGCGCCTGGTCACC | CGCTCCTGGAAGATGGTGATGG | 202 |
| *TNF-α* | NM_001278601 | GCTGAGCTCAAACCCTGGTA | CGGACTCCGCAAAGTCTAAG | 118 |
| *IL-1β* | NM_008361 | GCCTCGTGCTGTCGGACCCATAT | TCCTTTGAGGCCCAAGGCCACA | 143 |
| *MCP-1* | NM_011333 | TTGACCCGTAAATCTGAAGCTAAT | TCACAGTCCGAGTCACACTAGTTCAC | 110 |
| *TGF-β1* | NM_011577 | CCACCTGCAAGACCATCGAC | CTGGCGAGCCTTAGTTTGGAC | 91 |
| *Col-I* | NM_007742 | TAGGCCATTGTGTATGCAGC | ACATGTTCAGCTTTGTGGACC | 110 |
| *Fn* | NM_001276413 | CTGACTGGCCTTACCAGAGG | GTTGTCATGGCACCATTCAG | 288 |
| *ATG7* | NM_001379130 | TGCCAGAGGATTCAACAT | CATCAATAGGAAGACGACATC | 139 |
| *LC3B* | NM_026160 | TTATAGAGCGATACAAGGGGGAG | CGCCGTCTGATTATCTTGATGAG | 109 |
| *P62* | NM_011018 | AGGATGGGGACTTGGTTGC | TCACAGATCACATTGGGGTGC | 178 |
